# Explaining Individual Differences in Executive Functions Performance in Multilinguals: The Impact of Code-Switching and Alternating Between Multicultural Identity Styles

**DOI:** 10.3389/fpsyg.2020.561088

**Published:** 2020-10-23

**Authors:** Jeanine Treffers-Daller, Zehra Ongun, Julia Hofweber, Michal Korenar

**Affiliations:** ^1^Department of English Language and Applied Linguistics, University of Reading, Reading, United Kingdom; ^2^Department of Psychology and Human Development, University College London, London, United Kingdom; ^3^School of Psychology and Clinical Language Sciences, University of Reading, Reading, United Kingdom

**Keywords:** bilingualism, executive functions, inhibition, bilingual advantage, multicultural identity, code-switching, Turkish, Cyprus

## Abstract

This study sheds new light on the relative impact of switching between languages and switching between cultures on Executive Functions (EFs) in bilinguals. Several studies have suggested that bilingualism has a measurable impact on executive functioning, presumably due to bilinguals’ constant practice in dealing with two languages, or two cultures. Yet, the evidence on the relative contribution of culture and bilingualism to EFs is not well understood, because disentangling language, culture and immigration status is very difficult. The novelty of our approach was to keep the language pair and immigration status constant, whilst the cultural identity of participants was systematically varied, and measured at the individual level (not just at group level). Two groups of Turkish–English bilinguals, all adult immigrants to the United Kingdom, took part in the study, but one group (*n* = 29) originated from mainland Turkey and the other (*n* = 28) from Cyprus. We found that the bilinguals experienced smaller Conflict Effects on a Flanker task measuring inhibition, by comparison with monolingual British participants (*n* = 30). The key variable explaining EF performance variance at the individual level turned out to be bilinguals’ Multicultural Identity Style. In particular those who indicated that they attempted to alternate between different British and Turkish (Cypriot) identity styles were found to have shorter RTs on incongruent trials of the Flanker task. The two multicultural identity variables, Alternating and Hybrid Identity Styles, together explained 32% in RTs over and above Education, Working Memory and Nonverbal reasoning (overall explained variance 49%). Thus, the data provide strong evidence for the impact of culture on EFs. We suggest that, as a result of their daily practice in recognizing cultural cues which highlight the need to switch to a different cultural frame, multicultural bilinguals develop a heightened context-sensitivity, and this gives them an advantage over monolinguals in a Flankers task. Our approach, which draws on models from cross-cultural psychology, bilingualism and executive functioning, illustrates the importance of theory building in which sociolinguistic and cultural variables are integrated into models of EFs.

## Introduction

One of the most fascinating findings in the field of bilingualism is the fact that using more than one language in daily life can bring about advantages in Executive Functions (EF), that is the range of high-level control functions that support goal-directed behavior. However, initial findings which indicated that bilinguals are better than monolinguals at suppressing irrelevant information in non-linguistic tasks Bialystok (2001) were not always replicated. One reason for the conflicting findings is that there are different views of what EF are, and tasks are often impure in that they measure more than one skill and may tap different aspects of EF (Valian, 2015). Second, there are methodological differences between studies, which can make it difficult to compare results: these include differences in the choice of EF tasks (e.g., Simon Task versus Flanker Task; Poarch and Krott, 2019; Poarch and Van Hell, 2019), the issue of the ways in which different components of EF are measured, and sample size: as pointed out by Paap et al. (2017), using small samples increases the likelihood of a type I error or false positive. Third, in many studies, bilingual groups comprise speakers of a great variety of different languages. It is therefore not impossible that the great variability *within* bilingual groups obscured any of the intergroup differences *between* monolinguals and bilinguals. Any null results would then be due to noise and would thus reflect a type II error or false negative. As bilingualism covaries with cultural variables (Tran et al., 2019), a confound between language and culture compounds the problem. It is therefore important to try and disentangle the effects of these variables, which this article sets out to do.

While the debate about the existence of the “bilingual advantage” continues unabated, Prior and Gollan (2011) point out that in those studies where bilinguals were indeed found to outperform monolinguals on an EF task, it is not clear which particular characteristic of bilingualism was responsible for the effects. Costa et al. (2009) were probably the first to propose that bilinguals’ switching between languages and the need to monitor this behavior is at the heart of the bilingual advantage. These studies start from the assumption that inhibitory mechanisms involved in managing linguistic and non-linguistic tasks are shared, which leads to transfer effects. Neuroimaging evidence supporting this assumption demonstrates that there is indeed an overlap in brain networks involved in language selection and non-verbal task switching (Abutalebi and Green, 2007; Luk et al., 2011; De Baene et al., 2015). However, in recent studies such an overlap is further specified as being valid for bilinguals only (Anderson et al., 2018; Wu et al., 2019; Stasenko et al., 2020; Sulpizio et al., 2020).

Evidence for the role of code-switching as a source of the bilingual advantage was obtained by Hofweber et al. (2016), who showed that bilinguals’ EF performance is not only affected by how *frequently* bilinguals engage in code-switching but also by the specific *type* of intrasentential of code-switching they engage in. This indicates that a fine-grained approach which takes into account the different types of code-switching as distinguished by Muysken (2000, 2013) is needed. In addition, it is important to investigate to what extent code-switching and cultural variables covary in studies of EFs. As already noted by Peal and Lambert (1962) and Hilchey et al. (2015), it could be bilinguals’ wider experience with two cultures that gives them an advantage over monolinguals rather than their linguistic abilities in two or more languages. The few studies that have attempted to dissociate the effects of language and culture on EFs have produced contradictory results: some have found the effects of language to be stronger than those of culture (e.g., Yang et al., 2011; Barac and Bialystok, 2012), whilst others have found the opposite (Samuel et al., 2018) or found that the effects of culture were stronger on behavioral regulation/response inhibition while the effects of bilingualism were most visible in selective attention, switching and inhibition (Tran et al., 2019). Particularly interesting is the approach taken by Ye et al. (2016) who used the Flankers task developed by Wu and Thierry (2013) but administered it not with intervening words from two languages but with intervening pictures which were typical for either Chinese or English cultures: in the single culture block, all pictures were Chinese or British/American. In the mixed culture block, half of the pictures were Chinese and the other half were American or British. They found that high proficiency bilinguals had lower error rates than low proficiency bilinguals in the mixed culture block, but not in the single culture block. They conclude that “bi-cultural context ‘enhances’ proficient bilinguals’ cognitive performance” (Ye et al., 2016, p. 848). Because monolinguals were not included in the study, it is not clear whether the bilinguals in Ye et al. (2016) also had an advantage over monolinguals in their ability to switch between cultural frames.

In addition to the issue of culture, immigration status makes studying the cognitive effects of bilingualism complex. It is difficult to compare bilinguals who are immigrants (e.g., French immigrants in the United Kingdom) with monolinguals from the home country (e.g., French speakers from France) or the host country (e.g., British monolinguals in the United Kingdom), because bilingualism is then confounded with immigration status. As pointed out by Valian (2015), some researchers who have controlled for immigration status have found that bilinguals have an advantage over monolinguals, but such effects are not always repeated. Other studies which control for immigration status look at indigenous bilinguals only. Garraffa et al. (2017), for example, studied bilinguals who speak a regional minority language (Sardinian) in addition to the majority language (Italian) and compared these with monolingual speakers of Italian. They found, i.a., that bilinguals had better working memory skills. Because of the conflicting results in this field, we suggest that we need to take a more fine-grained approach toward cultural differences by measuring culture not just at the group level, by comparing immigrants from two different cultures, but at the individual level too, by adopting an individual differences approach to biculturalism.

As pointed out by Luk and Bialystok (2013), bilingualism is not a categorical variable. In a similar vein, we argue that culture is not a categorical variable either. Bilinguals do not belong to either one or the other culture. Instead, as Grosjean (2015, p. 575) argues, “bilinguals take part to varying degrees in the life of two or more cultures.” In other words, there are individual differences in the degree to which bilinguals are bicultural, and the ways in which bicultural individuals switch between, combine or blend elements of different cultures. We overlook these individual differences if we only measure culture at the group level. Interestingly, in the field of cross-cultural psychology, the ways in which bicultural individuals negotiate their cultures have received a great deal of attention in recent years. In their Transformative Theory of Biculturalism, West et al. (2017, p. 975) suggest that bilingual advantages may be “more reliably found” among bilinguals who are also bicultural, which confirms the findings of Ye et al. (2016). In other words, they suggest that biculturalism impacts on cognition, but what the impact consists of depends on the identity negotiation strategies chosen by the individuals. West et al. (2017) propose that the strategies bilinguals use to negotiate their identities include Hybridizing (“Synthesizing preexisting cultures into a new and distinct form by actively combining elements of both cultures”), and Frame switching (“Activating one of the two cultural systems in response to cultural context”)^1^. While the exact impact of the different strategies on cognition is not spelled out in great detail, the authors make some interesting predictions, namely that those engaging in switching between cultural frames need to monitor their context for cultural cues, such as an image depicting a scene characteristic of one of the two cultures, or situational cues, that is the arrival of a member of the other ethnic group, that alert them to the need to switch between cultural frames. Thus, Frame switching might lead to enhanced context-sensitivity. By contrast, the authors suggest that biculturals engaging in Hybridizing might increase the use of hybrid categories in social information processing (e.g., when Asian students combine Western individualistic values with collectivist values in their own personal values).

Ward et al. (2018, p. 1402) elaborate on the theory put forward by West et al. (2017). Ward et al. use the term “multicultural identity styles” for the strategies of Blending and Alternating that individuals use to manage multiple cultural identities (see section “Distinguishing between bilingualism and multiculturalism in studies of EFs” for more details). These correspond, by and large, to the categories of Hybridizing and Frame switching introduced by West et al. (2017).^2^

To the best of our knowledge, the work of West et al. (2017) and Ward et al. (2018) has not yet been used in studies of the effects of bilingualism and biculturalism on EF. The current project sets out to further explore the relative impact of code-switching and multicultural identity on EFs in adult Turkish–English bilinguals in the United Kingdom. Our approach is novel, not only because we measure multicultural identity at the group level as well as the individual level, but also because we keep the languages and immigration context constant but vary the cultural backgrounds of the participants, which allows us to disentangle the role of language and culture in ways that has not been possible so far.

We will first look at models of bilingual processing and EFs, and the available evidence regarding the effect of code-switching and multicultural identity on EFs, after which we will present the research questions, methods and findings of our study.

### Executive Functions and Models of Bilingual Processing

In their new model of EFs, labeled the unity/diversity framework, Miyake and Friedman (2012) propose that different EFs tap a common underlying ability, which they call Common EF. As inhibition correlates perfectly with this common core, for the purposes of the current study, we follow Valian (2015) who suggests the common factor should be labeled inhibition.

A key issue for researchers studying the link between EFs and bilingualism is that they need to account not only for the ability to inhibit words and task schemas from non-target languages but also for the fact that bilinguals can switch freely between languages in some contexts. In their Adaptive Control Hypothesis (ACH), Green and Abutalebi (2013) have therefore proposed that inhibitory control is not unitary across different contexts but adapts to the different demands placed upon it. These demands may differ depending on the contexts in which bilinguals find themselves: in *single language contexts*, bilinguals use one language exclusively in context A (e.g., at work) and another language in context B (e.g., at home), with very little code-switching between languages. In *dual language contexts*, by contrast, different languages are used with different interlocutors, so code-switching may take place but only between utterances (intersentential code-switching). Finally, in *dense code-switching contexts*, speakers freely mix both languages within one utterance (intrasentential code-switching). Competition between language task schemas differs by context in that the task schemas compete in the single and dual language mode, but co-operate in the dense language mode. More specifically, Green and Abutalebi distinguish between eight different control processes that are recruited to different degrees across the three contexts: the demands placed on inhibition and monitoring are greatest, for example, in the dual language context, and smallest in the dense code-switching context. These cognitive processes include (a) *goal maintenance*, that is the need to speak one language rather than another; (b) *interference suppression*: bilinguals need to inhibit irrelevant information from non-target stimuli in incongruent trials on e.g., a Flanker or a Simon Task; and (c) *conflict monitoring*: bilinguals also need to monitor when to inhibit particular task schemas or lemmas.

While the ACH makes testable explicit predictions about the relationship between code-switching and cognitive control, there are several issues with this model and its predictions regarding code-switching. First of all, it is based on a rather basic classification of code-switching, namely the distinction between intersentential code-switching (dual language contexts), and intrasentential code-switching (dense code-switching contexts). Thus, it treats code-switching as a categorical variable, whereas treating it as a more continuous variable by considering different “gradients” of intrasentential CS is more appropriate given what we know about the variability in code-switching patterns (Lai and O’Brien, 2020). As shown in Hofweber et al. (2016, 2020), these distinctions are relevant for bilinguals’ performance on EFs tasks.

Secondly, the ACH predicts that conflict monitoring and interference suppression (inhibition) are not recruited by bilinguals in dense code-switching contexts. While we agree that interference suppression is limited in dense code-switching contexts, Hofweber et al. (2016) argue that co-operation between languages in the dense context requires careful monitoring of the ways in which lemmas and task schemas from the two languages can be combined. Particularly when the grammars from the participating languages differ considerably, conflict monitoring skills need to be recruited to construe an utterance containing words from two languages. A more fine-grained dual control mode perspective (Hofweber et al., 2019) would suggest that intrasentential code-switching trains the types of EF recruited under conditions challenging conflict monitoring, whilst intersentential code-switching recruits global inhibitory processes to suppress the non-target language.

Thirdly, the ACH claims that in the dense code-switching context, speakers mainly rely on “*opportunistic planning*,” which means “making use of whatever comes most readily to hand in order to achieve a goal” (Green and Abutalebi, 2013, p. 519). However, if bilinguals in dense contexts mainly rely on opportunistic planning, the model predicts random variability in code-switching patterns in these contexts, because different bilinguals will have different words and structures at their disposal, and decisions on when to switch will be highly idiosyncratic. While bilinguals can be very creative in their code-switching, naturalistic code-switching data suggest that code-switching does not only depend on idiosyncratic choices, but is also influenced by the sociolinguistic practices of the speaker’s community. Code-switching patterns differ systematically depending on typological distance between languages and sociolinguistic factors such as depth of language contact and immigrant status (Muysken, 2000, 2013). Such regular patterns are more likely to result from gradual learning of conventional code-switching patterns that are typical for a particular community, so dense code-switching is not random, as was also pointed out by Green and Wei (2014). This does not mean that opportunistic planning does not exist, but that this process alone cannot account for the complexity of dense code-switching.

Clearly more evidence is needed regarding the effect of interactional contexts on EFs. Such evidence should come, first and foremost, from contexts where dense code-switching is a widespread discourse mode, that is in highly multilingual environments where several languages are commonly shared among speakers, as in Singapore (Ooi et al., 2018). In their study, Ooi et al. (2018) found that bilinguals who came from a dual language or dense code-switching context outperformed those from a single language context on a Flanker task, although correlations with self-reported code-switching behavior were not significant. Kang and Lust (2019) did look into actual code-switching behavior but did not find a link between code-switching and EFs among Chinese-English bilingual children. The authors do acknowledge that such effects might have been found if they had looked into different types of code-switching, as distinguished by Muysken (2000, 2013), to which we turn our attention now.

### Muysken’s (2013) Typology of Code-Switching

The framework proposed by Muysken (2013) distinguishes between (1) insertion of single words from language A (the societally dominant language) in a matrix structure of language B (the heritage language); (2) alternation, that is switching between longer stretches in language A and language B; (3) congruent lexicalization, where the grammars and lexicons of both languages are mixed, and (4) backflagging, where discourse markers from language B (the heritage language) are loosely attached to the structures in language A (the societally dominant language), as in (4). These represent examples of the different code-switching types for the language combination we investigated in this study, i.e., Turkish–English (1 = insertion, 2 = alternation, 3 = congruent lexicalization, 4 = backflagging). In the examples, English is in bold type face and Turkish in regular font. All examples were chosen from naturalistic data sets of Turkish–English code-switching except for (3), which is translated from Turkish–German.

(1) **Squirrelın** da iki dane **nutı** varıdı da (insertion of English nouns into a Turkish syntactic frame)
  Squirrel-GEN also two counts nut = 3SG COP-PAST also
  “The squirrel had two nuts as well.” (Aktuğlu and Sözüdoğru, 2011)
(2) Test yaptınız **near the phone**? (alternation)
  Test do-2ndPL near the phone
  “Did you test it near the phone?” (İssa, 2006)
(3) Aǧustos **is** iğrenç (congruent lexicalization)^3^
  August is disgusting
  “August is disgusting.” (Treffers-Daller, 2020)
(4) Haydi, **kettles come in handy^4^** (backflagging)
  Come on, kettles come in handy
  “Come on, kettles come in handy.” (Treffers-Daller, 2020)

According to Muysken (2000, 2013) speakers of typologically different languages are less likely to engage in congruent lexicalization than speakers of typologically similar languages. It was indeed very difficult to find unambiguous examples of congruent lexicalization for Turkish–English code-switching as these two languages differ widely from each other. However, Muysken’s model also predicts that heritage speakers with a long tradition of co-activating two languages will engage more in congruent lexicalization than recent immigrants, who mainly use insertion. It is therefore possible that some Turkish-speaking immigrants in the United Kingdom who have used English almost all their lives engage in this type of code-switching. Which of these four types of code-switching are covered by the term “dense code-switching” in the ACH is unclear. Some examples given by Green and Abutalebi (2013) appear to be insertions of single verbs from language A which are morphologically integrated into language B, while others contain a combination of insertions and alternations. We therefore assume that dense code-switching as used in the ACH refers to a variety of different intrasentential code-switching phenomena, while others (Green and Wei, 2014; Hofweber et al., 2016) use it as an equivalent of congruent lexicalization (see Table 1 for an overview). Because the term “dense code-switching” is ambiguous, in the current paper we use the term *intrasentential code-switching* to cover the four different types of code-switching within sentences proposed by Muysken (2013).

**TABLE 1:** Overview of terminology used to refer to intrasentential code-switching in models of bilingual speech processing.

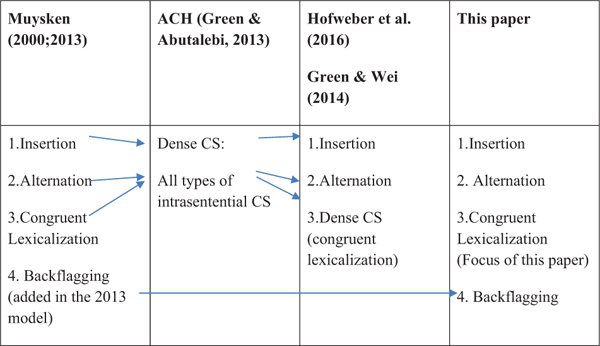

### Distinguishing Between Bilingualism and Multiculturalism in Studies of EFs

Studying the relationship between culture and EFs is more difficult than studying the relationship between code-switching and EFs, because of the lack of a theory or model of how linguistic and cultural factors interact in shaping EFs. In this paper we aim to make a contribution to theory creation in this field. We start from the assumption that in their everyday lives bilinguals may switch between cultures (West et al., 2017), as when bilinguals adopt different apology strategies depending on the cultural background and the linguistic profile of the interlocutors (Hatipoğlu, 2009). Second, we hypothesize that training in switching between cultures trains EFs in ways that are comparable to the training received by bilinguals who regularly switch between languages. This hypothesis is partly based on the suggestion that biculturals who engage in Frame switching need to consistently monitor their context for cultural cues that flag up the need to switch to a different cultural frame in ways that are comparable to switching between languages (West et al., 2017, p. 979). As monitoring is one of the EFs distinguished in Abutalebi and Green’s ACH, it is likely that switching between cultures recruits EFs. The hypothesis is also partly based on the study by Ye et al. (2016), who found that mixing cultures (or switching between cultures) enhances bilinguals’ cognitive performance.

Ward et al. (2018) use a slightly different terminology from West et al. (2017), and suggest that multicultural individuals can either try to blend different elements from each culture (*hybrid identity style*) or try to keep both identities separate and alternate between different identities (*alternating identity style*)^5^. As pointed out in the previous paragraph, these distinctions are relevant for the discussion about EFs, because bilinguals’ levels of identification with different cultures could impact on their propensity to keep their languages and cultures separate and inhibit one of these where the situation requires it. The degree to which bilinguals adopt multicultural identities and the type of identity they adhere to could constitute an important source of variability that has been neglected in studies of the relationship between bilingualism and EFs and might explain contradictory findings. Support for the idea that cultural identity impacts on a range of cognitive processes also comes from the field of creativity. Gocłowska and Crisp (2014, p. 217), for example, suggest that “compared to their more “homogenous” peers, dual-identity individuals, throughout their cultural adaptation experience, learn to alternate between their two identities, reconcile inconsistent values or cognitions, and broaden their self-definition.” This in turn, they claim, can lead to individuals becoming better at tasks challenging EFs.

While relatively little research is available about the link between EFs and identity in bilinguals, it is well known that code-switching is linked to identity (e.g., Myers Scotton, 1983), and that code-switching patterns are influenced by bilinguals’ attitudes and societal norms prevalent in their sociolinguistic environment (Poplack, 1988; Treffers-Daller, 1992), which will in turn shape individuals’ identities. Thus, we argue both code-switching and multilingual identities are shaped by bilinguals’ levels of engagement with their speech communities, as well as idiosyncratic variables from individuals’ personal backgrounds. This is why we investigate the matter both through group comparisons (stressing the speech community aspect) and by assessing individual differences (stressing the idiosyncratic aspects).

We hope that including analyses of code-switching as well as multicultural identity in one study will lead to a more in-depth understanding of the role of bilingualism and culture in EFs task performance than has been possible so far.

## The Current Study

The current study aims to contribute to the discussion about the bilingual advantage by comparing performance on EF tasks in two groups of Turkish-English bilinguals and one group of monolingual speakers of English. It builds on existing research on the relationship between intrasentential code-switching and EFs by Hofweber et al. (2016, 2019), which had shown positive correlations between congruent lexicalization and conflict-monitoring amongst bilinguals who used two typologically similar languages (German and English). Our study differs from that of Hofweber et al. (2016, 2019) in that we explore bilinguals who speak typologically different languages (Turkish and English). The novelty of the current study resides in the fact that we study the impact of culture on EFs by keeping the languages and immigration context constant but varying the cultural backgrounds of the participants: both groups consisted of first-generation immigrants to the United Kingdom, but one group originated from mainland Turkey, and the other group from Cyprus. In other words, in our study we investigate two bilingual groups which differ in terms of their socio-cultural identity but speak the same two languages, which allows us to tease apart the impact of language and culture on EFs.

The hypotheses formulated for the current study were as follows. Our study follows on from Hofweber et al. (2016, 2019, 2020), who confirmed Muysken’s (2000) observation that code-switching patterns differ as a function of sociolinguistic environments and that contexts with more established multilingual traditions favor intrasentential code-switching, in particular congruent lexicalization. Therefore, on the basis of the fact that Cyprus-born bilinguals have a longer tradition of contact with English than the Turkey-born bilinguals our hypothesis was that (a) the Cyprus-born bilinguals would engage more in congruent lexicalization and, (b) that Cyprus-born bilinguals would have higher levels of hybrid identity styles than the Turkey-born bilinguals who were predicted to have lower levels of hybrid identity styles.

As for the between group differences in EFs, we based our study on the assumption that code-switching is at the heart of the bilingual advantage (Costa et al., 2009), and predicted (a) that the two bilingual groups would outperform the monolinguals on an EFs task and (b) that the Cyprus-born bilinguals would outperform the Turkey-born bilinguals, as a result of the enhanced training in EFs they received through their practice with congruent lexicalization. However, we also formulated a competing hypothesis, derived from Green and Abutalebi’s (2013) ACH, namely that Inhibition is *not* trained in bilinguals who practice intrasentential code-switching. This model predicts that those frequently engaging in this type of code-switching (in particular congruent lexicalization) would underperform by comparison with those who do this less often.

With respect to the individual differences in EFs we formulated two competing hypotheses, namely (a) if *language* is the key determining factor behind the bilingual advantage, code-switching practices would explain variance over and above non-linguistic variables that have often been found to covary with EFs (Education, Age, Working memory and Non-verbal reasoning) as well as over and above measures of Multicultural Identity. Conversely, (b) if *culture* is the key factor, measures of Multicultural identity would be the key explanatory variable (over and above other non-linguistic and linguistic variables, including code-switching).

Thus, the contribution of multicultural identity and code-switching practices to EFs was explored through group comparisons as well as through an individual difference approach assessing the predictors of Inhibition.

### Methods

To test our hypotheses, a mixed design was used, with Language Group (LG) as the between group variable, with three levels: Turkish bilinguals (TBLs), Cypriot bilinguals (CBLs) and monolingual English speakers (MLs). Within group variables were code-switching patterns, Multicultural identity styles (HIS and AIS) and two variables measuring Inhibitory Control, namely the Conflict Effect and performance on Incongruent Trials on a Flanker task. After analyzing the between group differences, we investigated to what extent linguistic and non-linguistic variables could explain variance in EFs across the three groups and within each group.

#### Participants

Participants were Turkey-born (*n* = 30) and Cyprus-born adult bilinguals (*N* = 30) and monolingual adult speakers of English (*n* = 31). The data for the latter were collected as part of a separate project on code-switching and EFs led by Hofweber et al. (2016, 2019). All participants were residents in the South East of the United Kingdom, and all from middle class backgrounds (see Table 2 for further details). Four informants from the original pool were excluded from further analyses because of outliers on EFs tasks^6^.

**TABLE 2:** Overview of participant characteristics (before matching).

		Group 1 TBL (*N* = 29)			Group 2 CBL (*N* = 28)		Group 3 ML (*N* = 30)		
	*M*	*SD*	*M*		*SD*	Mean	*SD*	*F*	*p*	
Age		32.48	7.95	25.5	3.98	32.33	10.06	7.96	0.001	1 = 3; 2 < 1,3
Edu		3.00	0.85	2.64	1.03	3.87	0.63	16.24	< 0.001	1 = 2, 3 > 1,2
Gen		1.55	0.51	1.54	0.51	1.53	0.51	0.11	0.99	n.s.
NVr		−0.44	0.81	0.35	1.01	−0.11	0.95	5.23	0.01	1 = 3; 2 = 3, 1 < 2
WMfZ		−0.64	0.82	0.72	0.72	0.77	0.95	19.85	<0.001	3 > 2 > 1
WMbZ		−0.46	0.88	0.67	0.60	−0.20	1.12	12.61	<0.001	1 = 3, 2 > 1,3
TyU		29.69	8.26	22.57	3.86	n.a.		17.81	<0.001	1 > 2
EyU		21.28	7.23	18.93	4.67	n.a.		2.10	0.15	1 = 2
Tnst		2.76	2.17	3.07	0.66	n.a.		0.54	0.47	1 = 2
Enst		10.83	8.12	4.63	3.76	n.a.		13.11	0.001	2 < 1
Esr		5.72	0.92	6.06	0.60	n.a.		2.80	0.10	1 = 2
Tsr		5.75	1.89	5.47	0.91	n.a.		0.49	0.48	1 = 2
MixFa		4.17	2.47	1.11	0.32	n.a.		42.60	<0.001	1 > 2
MixFr		3.41	2.03	1.11	0.32	n.a.		35.42	<0.001	1 > 2
MixW		3.48	2.61	1.07	0.26	n.a.		23.59	<0.001	1 > 2
Twl		4.76	2.21	3.14	1.76	n.a.		9.27	0.004	1 > 2
Bwl		4.10	2.29	5.86	1.01	n.a.		13.84	<0.001	2 > 1

*Age, age in years; Edu, education, 1 = low, 5 = high; Gen, gender; NVr, non-verbal reasoning; WMfZ, working memory, forward digit span (Zscore); WMbZ, working memory, backward digit span (Zscore); TyU, years of use of Turkish; EyU, years of use of English; Tnst, Turkish onset; Enst, English onset; Esr, English self-rating; Tsr, Turkish self-rating; MixFa, language mixing in the family; MixFr, language mixing with friends; MixW, language mixing with coworkers; Twl, Turkish way of life; Bwl, British way of life.*

The varieties of Turkish spoken by Turks and Cypriots, although mutually easily comprehensible, are clearly distinct at the levels of vocabulary, grammar and pronunciation, and also because of influence from Greek and English in Cypriot Turkish (see Adalar and Tagliamonte, 1998; İssa, 2006), although in writing only the Turkish standard variety is used. Cypriots speak both varieties, and Standard Turkish is widely used on the island: after the Turkish invasion of the island in 1973, mass migration from the mainland to the island took place, Turkish TV channels can be received in Cyprus and the universities attract substantial numbers of students from Turkey every year. Standard Turkish has also been the official language of Northern Cyprus since 1985.

According to Sirkeci and Esipova (2013), there are between 180,000 and 250,000 Turkish-speaking immigrants in the United Kingdom. These belong to three main groups: Turks, Cypriots, and Kurds. The vast majority of the Turkish and Turkish Cypriot communities are based in London, with smaller numbers living in Birmingham and Manchester. The 2011 census data show that most of the immigrants were born in Turkey (93,916) and a smaller group in Northern Cyprus (3,026), but these figures do not include immigrants from the second and third generations, many of whom were born in the United Kingdom.

The history of immigration of Turkish-speaking groups to the United Kingdom shows that a first wave of Turkish Cypriots arrived in the 1950s as a result of hostilities between the Greek and Turkish communities. A second wave of immigrants from Cyprus came after the Greek coup and the invasion of Cyprus by Turkey in 1974. Turks from the mainland arrived in the United Kingdom from the late 1970s onward, and in particular after the military coup in the 1980s, so considerably later than the Cypriots. The latter chose the United Kingdom because of the historic ties between the United Kingdom and Cyprus: the island had been part of the British Empire since the late 1800s and was a Crown colony until 1960. English is increasingly used for communication across the two communities, as well as more widely in commerce, tourism and education. Therefore it is an integral part of the daily lives of many Cypriots and very present in the linguistic landscape (Themistocleous, 2018), much more than in mainland Turkey.

The available literature suggests that code-switching is indeed practiced in online platforms among Turkish–English bilinguals (Yirmibeşoǧlu and Eryiǧit, 2018) and also among Turkish–English bilinguals in the US (Koban, 2013, 2016). Linguistic analyses of code-switching among Cyprus-based Cypriots show that there is intergenerational variability in that younger, British-born Cypriots speak more English (and identify more with English) than older Cyprus-born Cypriots and the younger ones switch more from Turkish to English than vice versa (Adalar and Tagliamonte, 1998; İssa, 2006; Aktuğlu and Sözüdoğru, 2011). Interestingly, Koban (2013, 2016) reveals that many Turkish–English bilinguals admit using code-switching in daily life, whilst holding negative attitudes toward this behavior.

For the purposes of the current study it is also important that Turkey-born and Cyprus-born bilinguals have clearly distinct identity profiles. Psaltis and Cakal (2016) note that in Cyprus the two communities remain largely segregated, with little interaction between them. According to Sirkeci et al. (2016, p. 167), this is also the case for the different Turkish-speaking immigrant communities in the United Kingdom, which differ from each other “in their lifestyles, experiences, ideas, feelings, hopes and expectations.” In addition, the authors suggest these groups “have been observed living in different ethnic, ideological, cultural and religious communities for decades” (Sirkeci et al., 2016, p.4). The lack of contact between both groups is likely due to the fact that many Turkish Cypriots report a high level of “perceived symbolic threat,” that is a threat to values and norms of the Turkish Cypriots posed by mainland Turks living in Cyprus (Cakal, 2012, p. 5). According to Psaltis and Cakal (2016) these individuals are generally referred to as “settlers” by Greek Cypriots and by Turkish Cypriots as “immigrants.” Turkish Cypriots also feel that their group esteem as Turkish Cypriots is undermined by those from the mainland and they perceive Greek Cypriots as threatening to their political and economic resources (Cakal, 2012, p. 5).

In summary, the Turkish Cypriots and the Turks from mainland Turkey constitute two clearly distinct sociocultural groups, although they share the same language, and those living in the United Kingdom also share immigrant status. This combination of variables makes these groups very interesting for a study which aims to fill a gap in our understanding of the relationship between language, culture and EFs.

Table 2 reveals that there are significant differences between the three groups on most non-linguistic variables (except gender) and some linguistic variables, including language mixing, and cultural variables, such as evaluations of the Turkish and the British ways of life. We used different techniques to control for key non-linguistic and linguistic variables that have an impact on EFs. We first carried out a mixed ANCOVA controlling for Education, Age, Working memory, and Non-verbal reasoning. This was followed by a series of univariate analysis with RTs from the Flanker task as the dependent variable (see data analysis for further details). To test the robustness of the effects obtained, we carried out a second series of analyses, for which we matched informants from the three groups at group level by excluding those informants whose scores on the key independent variables exceeded 1.4 SD (in either direction). For Working Memory and Non-verbal reasoning this criterion was not enough to ensure groups were matched, and therefore for these variables we excluded anyone with scores exceeding 1 SD in either direction (see Table 3 for details). While this meant a drastic reduction in the number of informants from 87 to 31, it was important to establish whether any effects which were found in the previous analysis would still obtain in analysis where informants were carefully matched at group level. In the second analysis the differences between the groups on the above variables were no longer significant, except for the reported frequency of language mixing. We again followed up with a series of univariate analyses as was done for the data set with all informants.

**TABLE 3:** Comparison of groups of informants after matching at group level.

		Group 1 TBL (*N* = 14)		Group 2 CBL (*N* = 11)		Group 3 ML (*N* = 9)				
	*M*	*SD*	*M*	*SD*	Mean	*SD*	*F*	*p*	
Age	28.21	5.48	27.82	3.57	27.22	8.41	0.08	0.93	
Edu	3.29	0.47	3.18	0.40	3.56	0.53	1.67	0.20	
Gen	1.5	0.519	1.73	0.467	1.44	0.527	0.938	0.40	
VPuZ	−2.08	0.31	0.10	0.44	−0.05	0.42	1.96	0.54	
DSfZ	−3.52	0.71	−3.12	0.83	0.21	0.66	1.78	0.19	
DSbZ	−2.37	0.76	−3.13	0.47	−0.20	0.44	2.97	0.07	
TyU	25.43	6.53	24.73	3.80	n.a.		0.100	0.76	
EyU	17.93	7.83	22.09	3.86	n.a.		2.60	0.12	
Tnst	2.64	1.95	3.27	1.01	n.a.		0.95	0.34	
Enst	8.36	6.74	3.27	1.01	n.a.		0.79	0.38	
Esr	5.89	0.98	6.23	0.75	n.a.		0.87	0.36	
Tsr	5.55	1.97	5.91	0.83	n.a.		0.31	0.58	
MixFam	4.43	2.59	1.09	0.30	n.a.		17.87	0.001**	1 > 2
MixFr	2.57	1.99	1.09	0.30	n.a.		5.93	0.023*	1 > 2
MixW	3.00	2.83	1.09	0.30	n.a.		4.92	0.037*	1 > 2
Twl	4.21	2.23	4.27	1.68	n.a.		0.01	0.94	
Bwl	4.50	2.25	5.64	0.92	n.a.		2.47	0.10	

*See Table 2 for explanation of abbreviations.*

#### Instruments

We used a Flanker task to measure inhibitory control because of its task purity (Costa et al., 2008). Participants were shown rows of five arrows and had to press a key to indicate the direction of the central arrow. In half of the trials all arrows faced in the same direction (congruent condition) and in the other half the middle arrow face in the opposite direction (incongruent condition). The difference between the reaction times (RTs) for these two types of trials is known as the Conflict Effect. At the start of each trial participants saw a fixation cross for 200 ms, followed by the 1000 ms stimulus presentation with a 1500 ms response time. Inter trial intervals were randomly varied (jittered), and varied in length from 200 to 3000 ms, as in Hofweber et al. (2016, 2019).

Crucially, the Flanker task was adapted to create a context challenging conflict-monitoring (Costa et al., 2009). Our Flanker task consisted of 48 congruent and 48 incongruent trials (preceded by six practice trials), presented in random order. This manipulation of the trial split required participants to continuously switch between congruent and incongruent trials, which generated a context challenging conflict-monitoring and thus challenged the EFs processes involved in dense forms of code-switching, especially congruent lexicalization. Our Flanker task was identical to the one used by Hofweber et al. (2016, 2019).

#### Code-Switching Frequency Task

We developed a 98-item frequency judgment task based on Hofweber et al. (2016) containing different types of Turkish-English CS as distinguished by Muysken (2013). There were fourteen examples per code-switching type (seven from Turkish to English and seven from English to Turkish), as well as fourteen monolingual control sentences (seven in each language), which consisted of translations of code-switching examples in the task. It also contained fourteen examples of mixed verbal compounds, which were not used for the current study. Utterance length was controlled by shortening examples to ten syllables. Two versions of the task were created (a Standard Turkish and a Cypriot Turkish version) because examples in Standard Turkish might not sound authentic to speakers of Cypriot Turkish. The switches were presented in random order, in oral form through headphones with support of the written form on a PPT slide (see Hofweber et al., 2019, for further details). Respondents were asked how frequently they encountered in their environment sentences such as those presented in the task. They were not asked whether they used these themselves because code-switching is a stigmatized form of language behavior in many communities, which means that respondents would be reluctant to admit producing sentences with intrasentential code-switching. Following Onar Valk and Backus (2013), we asked participants about “frequency” rather than “acceptability” of sentences to avoid participants referring to norms that are prevalent in a monolingual mode rather than in a bilingual mode. As shown in Hofweber et al. (2019), there is evidence that answers to a receptive code-switching frequency task correlate to bilinguals’ productive use of code-switching. Participants answered on a Visual Analog Scale (VAS) (Llamas and Watt, 2014), which consisted of a ten centimeter horizontal line on which the endpoints were labeled on the left as “never” and on the right as “always,” which allows for collecting more subtle answers than would be possible with a Likert scale. Scores on the VAS ranged from 0 to 100.

#### Multicultural Identity Styles Scale

Participants’ affinity with both cultures was measured with Ward et al.’s Multicultural Identity Scales, which tap into Alternating Identity Styles (AIS) and Hybrid Identity Styles (HIS). Statements such as “*I am British in a Turkish way*” represented the HIS and “*I can be British or Turkish depending on the circumstances*” represented the AIS (see Table 4 for all statements). Two versions were created of each questionnaire for use in the two different communities. In the version for Cypriot participants *Cypriot* replaced *Turkish*. The 20 statements were presented in random order and participants indicated their answers on a VAS, with endpoints indicating “not at all true of me” (on the left) to “completely true of me” (on the right). Again scores on the VAS ranged from 0 to 100.

**TABLE 4:** Summary of exploratory factor analysis results (Pattern matrix) of the Multicultural Identity Styles Scales.

	Identity statements (Hybrid versus Alternating identity Styles)	Factor 1	Factor 2
1A	I alternate between being British and Turkish depending on the circumstances		0.701
2H	The British and Turkish in me form one: I am a British Turk	0.914	
3H	I am British in a Turkish way		0.698
4A	I can be British or a Turkish depending on the circumstances		1.024
6A	I am very British with my family compared with other people	0.581	0.352
7A	Who I am depends on the social context	0.834	
9H	I am a “mélange” of Turkish and British	0.879	
10H	I see myself as a culturally unique mixture of British and Turkish	0.857	
11A	Some situations make it hard to be British and Turkish at the same time.		0.871
12H	For me, being British and being a Turkish are intermingled	0.309	0.6
13H	For me, being British and being a Turkish come together in a culturally novel way.	0.843	
14A	I have a Turkish private self and a British public self	0.466	0.374
15H	I am a blend of British and Turkish	0.8	
17A	I am Turkish at home and British at school/work	0.704	

*Statements marked A belong to the Alternating Identity Styles, and those marked H to the Hybrid Identity Styles.*

#### Language History Questionnaire

Li et al.’s (2014) Language History Questionnaire (LHQ 2.0) was used to collect data about respondents’ experience with Turkish and English as well self-ratings, and information about cultural differences. Participants took the Turkish translation that was available on the website of Hongkong Polytechnic University^7^.

### Tasks Assessing Fluid Intelligence/Cognitive Background Variables

Ravens’ progressive matrices (Raven and Raven, 2003), which is a pattern matching task widely used to measure non-verbal reasoning. Because the Turkish–English participants were part of a larger project which also included creativity tasks, they took a different non-verbal reasoning task which was more closely aligned with the construct of intelligence as defined in the Cattell-Horn-Carroll model (McGrew, 2009). They were administered the Visual Puzzles task (18 items) from the Wechsler Adult Intelligence Scales IV (WAIS IV) (Wechsler, 2008). Both groups also took a Forward and Backward Digit Span task (12 items each). For the Visual Puzzles, the participants were given a picture of a completed puzzle and needed to select pictures of three pieces from a total of six that make it possible to reconstruct the puzzle. In order to be able to compare the results of the Ravens Task and the Visual Puzzles task, we computed *Z*-scores of each task per group, and used these *Z*-scores for further analyses. The forward and backward Digit Span tasks consisted of six levels, with two items for each level, ranging from two to seven digits.

### Procedure

The study was part of a larger project in which two additional tasks which were not used in the current study were administered (a creativity task and a task switching task). Except for two tasks, which were counterbalanced across groups, the tasks were taken in the following order: (1) Creativity task, (2) Flanker task, (3) Task switching, (4) Verbal and non-verbal reasoning, (5) Code-switching frequency task, (6) Multicultural Identity Styles Scales and (7) Language History Questionnaire. The non-verbal EFs tasks (Tasks 2 and 3) were counterbalanced across groups, so that 15 participants in each group first took the Flanker task, followed by the Task Switching task and the remaining 15 took these tasks in the opposite order. All participants took the tasks individually in the presence of the second author, who is a native speaker of Turkish.

### Data Analysis

Error trials on the Flanker task were excluded from further analyses (2.34% of the responses). As in Hofweber et al. (2016), outlier responses deviating by more than 3 SDs from the mean for each participant were trimmed separately for congruent and incongruent trials. This procedure eliminated 2.67% of the data. We then carried out an exploratory analysis of the data to establish whether there were any extreme values at group level. Four informants whose scores were identified as extreme values by SPSS 24 were removed.

The accuracy scores were at ceiling (congruent *M* = 47.29, *SD* = 1.14; incongruent *M* = 46.26, *SD* = 1.29), and therefore not used for further analyses. The RTs were normally distributed after removal of outliers. This was the case for congruent trials (KS = 0.061, *df* = 87, *p* = 0.200), incongruent trials (KS = 0.061, *df* = 87, *p* = 0.200) and the Conflict Effect (KS = 0.079, *df* = 87, *p* = 0.200), which is computed as the difference in RTs on congruent and incongruent trials. As Valian (2015) recommends trying out different procedures for computation of measures of inhibition and monitoring, we also computed a Proportional Score by dividing the Conflict Effect by the RT for the congruent trials. This makes it possible to take into account individual differences in RTs that are otherwise ignored. The scores on the Proportional Score were also normally distributed (KS = 0.071, *df* = 87, *p* = 0.200).

The reliability coefficients for the CSFT (Cronbach’s α = 0.922, 6 items) and the MISS (Cronbach’s α = 0.957, 14 items) were high. A principal component analysis was carried out on the mean scores for the six variables within the CSFT (the means for four types of intrasentential code-switching, intersentential code-switching and monolingual Turkish and English sentences). The Kaiser-Meyer-Olkin measure verified the sampling adequacy for the analysis, KMO −0.839, meritorious according to Hutcheson and Sofroniou (1999). An initial analysis was run to obtain eigenvalues for each factor in the data. A two-factorial solution was found, which explained 92.26 percent of the variance. Any factor loadings lower than 0.3 were suppressed (Field, 2013). The data in Table 5 show the factor loadings after rotation. Oblique rotation (Oblimin with Kaiser normalization) was chosen because the factors cannot be assumed to be independent. The items that cluster on the same factor shows that factor 1 represents perceptions of intrasentential code-switching frequency and factor 2 perceptions of the frequency of monolingual sentences. Contrary to expectations, the four different types of intrasentential code-switching did not load on to different factors, which probably means that participants did not perceive these as fundamentally different. Interestingly, switching between sentences (intersentential code-switching) loaded onto the same factor as monolingual sentences, so was perceived as more similar to monolingual sentences than to switching within utterances. The different types of intrasential code-switching were not normally distributed so we log transformed the four categories using Log10 and found that INS (KS = 0.080, *df* = 57, *p* = 0.200), ALT (KS = 0.074, *df* = 57, *p* = 0.200), BFL (KS = 0.100, *df* = 57, *p* = 0.200) and CLX (KS = 0.105, *df* = 57, *p* = 0.180) were all normally distributed after transformation.

**TABLE 5:** Exploratory factor analysis of the CSFT (Pattern matrix).

Variable	Factor 1	Factor 2
Insertions total	0.896	
Alternations total	0.926	
Congruent lexicalization total	0.984	
Backflagging total	0.975	
Intersentential CS total		0.883
Monolingual English		1.007
Monolingual Turkish		0.942

A principal axis factor analysis was also conducted on the fourteen items of the Multicultural Identity Styles Scales with oblique rotation (Oblimin with Kaiser Normalization). Sampling adequacy was verified with the Kaiser-Meyer-Olkin measure (KMO = 0.916, which is “marvelous” according to Hutcheson and Sofroniou, 1999). The initial analysis showed that there were two factors in the data which together explained 72.38% of the variance. Table 4 shows the factor loadings after rotation. Factor loadings smaller than 0.3 were suppressed as recommended in Field (2013). As six of the seven of statements which purportedly tapped Hybrid Identities loaded onto the first factor, and five of the seven statements which measure Alternating Identities on the second, we labeled the first factor “Hybrid Identities” and the second one “Alternating Identities.” We then computed the mean of all variables which loaded strongly on Factor 1, and repeated this for those loading on Factor 2, and used these new mean AIS and HIS scores for further analyses.

## Results

We will first present the results of the code-switching and identity tasks, after which we will give an overview of the differences between bilinguals and monolinguals in the Flanker task. Finally, we will explore explanations for the variance in Flanker task performance.

### Bilinguals’ Code-Switching Practices and Multicultural Identity Styles

**FIGURE 1:**
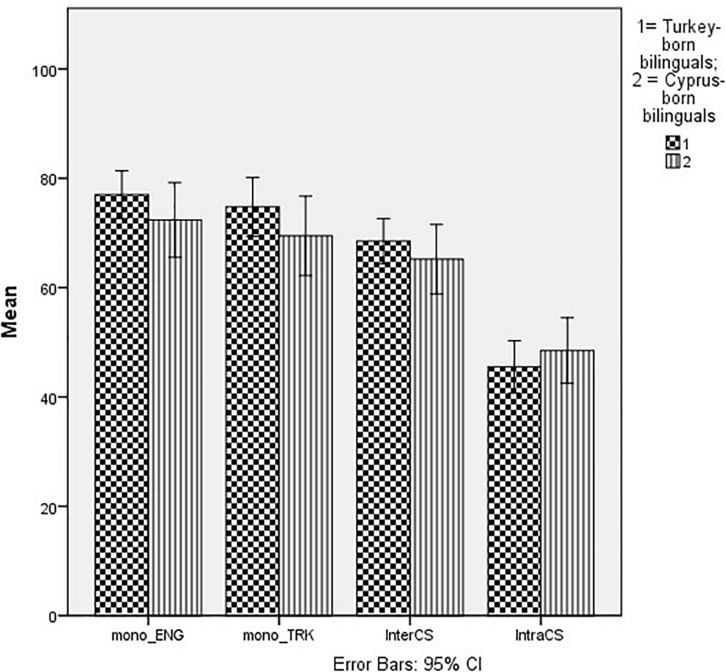
Frequency of code-switching and monolingual utterances by Group.

Figure 1 gives an overview of the frequency with which respondents claimed to encounter monolingual English and Turkish sentences, as well as intersentential and intrasentential code-switching. This Figure shows that sentences with intrasentential code-switching were claimed to be heard least often. Because the monolingual sentences and the intersentential code-switching variables were not normally distributed, a Friedman’s ANOVA was used to test whether intrasentential code-switching (language mixing) was used less frequently than other categories. This was the case for both groups (TBLs, χ*^2^* = 63.29, *df* = 3, *p* < 0.001; CBLs, χ*^2^* = 41.70, *df* = 3, *p* < 0.001). This was followed up with pairwise Wilcoxon tests. These show that among TBLs language mixing was indeed less frequent than monolingual English sentences (χ^2^ = 4.70, *p* < 0.001), less frequent than Turkish monolingual sentences (χ*^2^* = 4.70, *p* < 0.001) and also less frequent than intersentential code-switching (χ*^2^* = 4.70, *p* < 0.001). Among CBLs, comparisons with monolingual English sentences (χ^2^ = 4.486, *p* < 0.001), with monolingual Turkish sentences (χ*^2^* = 4.30, *p* < 0.001) and with intersentential code-switching (χ*^2^* = 4.42, *p* < 0.001) were all significant too.

The Figure also shows that intrasentential code-switching is slightly more frequent among CBLs. Further analyses revealed that this is due to the marginally higher frequency of congruent lexicalization among CBLs (*t* = 3.61, *df* = 1,55, *p* = 0.063; η*^2^_*p*_* = 0.06) and in particular congruent lexicalization from Turkish to English, as in (3), where English function words appear in a sentence which consists of Turkish words and homophonous diamorphs (*t* = 5.0, *df* = 1,55, *p* = 0.050, η*^2^_*p*_* = 0.07). Congruent lexicalization in the opposite direction (from English to Turkish) was not significantly different (*t* = 2.45, *df* = 1,55, *p* = 0.123, η*_*p*_* = 0.04). In all cases effect sizes were very small. There were no significant differences between the groups with respect to the other code-switching types.

The results of the CSFT contrast with those of the respondents’ self-reported language mixing behavior. Four questions from LHQ asked respondents to indicate on a seven-point scale (1 = never, 7 = always) how frequently they mixed languages in normal conversations in different domains of life. The mean rank of the scores on these questions is much higher for TBLs (mean rank = 40.19) than for CBLs (Mean rank = 17.41)^8^, and the difference between these two is significant (Mann–Whitney *U*-test, U = 81.50, *p* < 0.001) with a strong effect size (*r* = 0.72). Interestingly, the results from the CSFT and the self-reported mixing behavior do not correlate. Although it is not clear at this point why these two sources of information do not correlate, we know from the academic literature on Turkish–English code-mixing that it is frequent among Cypriots in the United Kingdom (e.g., İssa, 2006) as well as in Cyprus (Adalar and Tagliamonte, 1998). Therefore, it seems that the Cypriots are under reporting their code-switching behavior. Because code-mixing is stigmatized among some groups of Turkish–English bilinguals (see also Koban, 2016), we will assume these scores reflect attitudes toward code-mixing rather than actual frequencies.

As has already been shown in Table 1, there are also some interesting cultural differences between the two groups: Perceptions of the Turkish Way of life are more positive among TBLs (with a moderate effect size: *r* = −0.40), while perceptions of the British Way of Life are more positive among CBLs (with a moderate effect size, *r* = 0.36). Further information about cultural differences between the groups can be found in the results from the MISS. The mean rank for the HIS is 18.29 for TBLs and 40.09 for CBLs (Mann–Whitney *U* test, *U* = 716.000, *p* < 0.001), with a large effect size (*r* = 0.66). For the AIS, the mean ranks are 17.10 for TBLs and 41.32 for CBLs (Mann–Whitney *U* test, U = 751.000, *p* < 0.001, with a large effect size, *r* = 0.73). Self-reported code-switching frequency correlates positively (*r*_*s*_ = 0.54, *p* < 0.001) with respondents’ views of the Turkish way of life, and negatively with their views of the British way of life (*r*_*s*_ = −0.49, *p* < 0.001). There was also a strong negative correlation between the British and Turkish Ways of Life (*r*_*s*_ = −0.74, *p* < 0.001), and mid strength negative correlations between self-reported mixing and HIS (*r*_*s*_ = −0.41, *p* < 0.001) and AIS (*r*_*s*_ = −0.58, *p* < 0.001). However, the results from the CSFT did not correlate with the variables measuring British and Turkish Ways of Life.

Finally, the results on the CSFT did not correlate with the scores on the Flanker task, but there were mid strength correlations between *reported* intrasentential code-switching behavior (as measured with the LHQ) and the Flanker task in that those who reported to use more language mixing had longer RTs on incongruent trials on the Flanker task.

In summary, we found that CBLs engaged slightly more in congruent lexicalization, and had more pronounced Hybrid Identity styles than TBLs, although they also had more pronounced Alternating Identity styles, which was unexpected. As there was a stronger negative correlation between AIS and self-reported mixing than between HIS and self-reported mixing, it seems that those who identify more with AIS, and are therefore more likely to see themselves as having two separate cultural identities, are particularly negative about language mixing.

### Group Differences in EFs Performance

An overview of the descriptive results of the RTs for congruent and incongruent trials, the conflict effect and a proportional RT score is given in Table 6. A mixed design ANOVA was used to investigate whether monolinguals and bilinguals differed from each other with respect to RTs for congruent and incongruent trials on a Flanker task. As the groups differ from each other on a number of key non-linguistic variables (Non-verbal intelligence, Age, Education and Working memory), we first conducted an ANCOVA with Congruence as the repeated measures dependent variable (two levels: incongruent RTs and congruent RTs), Group as the between subjects factor (three levels: Turkish bilinguals, Cypriot bilinguals and monolinguals) and four covariates: Age, Education, Non-verbal reasoning (Visual puzzles, log10 transformed), and Working Memory (Sum of forward and backward digit span, log10 transformed). Levene’s Test of the Equality of Error variances was not significant [*F*(1,80) = 1.77, *p* = 0.176].^9^

**TABLE 6:** Mean RTs on the Flanker task per group.

Groups		Congruent RTs	Incongruent RTs	Conflict RTs	Proportional score
1	Mean	475.23	517.70	42.47	0.09
	Standard deviation	17.29	22.60	15.88	0.03
2	Mean	465.42	488.35	22.92	0.05
	Standard deviation	25.80	15.43	21.95	0.05
3	Mean	462.71	521.46	58.76	0.13
	Standard deviation	33.16	36.88	17.41	0.04
Total	Mean	467.76	509.55	41.80	0.09
	Standard deviation	26.58	30.30	23.50	0.05

The ANCOVA results showed that there was a significant main effect of Congruence, in that participants were faster on congruent trials than on incongruent trials [*F*(1,80) = 26.59, *p* = 0.001, η*^2^_*p*_* = 0.25]. There was no main effect of Group [*F*(2,80) = 0.50, *p* = 0.61, η*^2^_*p*_* = 0.01], but there was a significant interaction between Congruence and Group [*F*(2,80) = 24.65, *p* < 0.001, η*^2^_*p*_* = 0.38]. Two covariates were significantly related to the dependent variable: Non-verbal reasoning [*F*(1,80) = 6.56, *p* = 0.01, η*^2^_*p*_* = 0.08] and Age [*F*(1,80) = 13.50, *p* < 0.001, η*^2^_*p*_* = 0.14].

Because of the strength of the interaction between Congruence and Group we explored the intergroup differences in Congruence further by carrying out a series of univariate analyses, with four different dependent variables: congruent RTs, incongruent RTs, Conflict Effect and Proportional Score. The between groups variable was Group and the same covariates were included in the model as before.

We first ran an ANCOVA with Congruent RTs as the dependent variable. In this model the there was no main effect of Group [*F*(2,80) = 2.50, *p* = 0.09, η*^2^_*p*_* = 0.06]; only Age [*F*(1,80) = 10.62, *p* = 0.002, η*^2^_*p*_* = 0.12] and Non-verbal reasoning [*F*(1,80) = 6.96, *p* = 0.001, η*^2^_*p*_* = 0.08] were significant. By contrast, in the next model, with Incongruent RTs as the dependent variable, there was a significant main effect of Group [*F*(2,80) = 5.26, *p* = 0.007, η*^2^_*p*_* = 0.12]. A *post hoc* analysis showed that CBLs and MLs were significantly different from each other (*p* = 0.006). However, Age [*F*(1,80) = 12.57, *p* = 0.001, η*^2^_*p*_* = 0.14] and Non-verbal reasoning [*F*(1,80) = 4.48, *p* = 0.04, η*^2^_*p*_* = 0.05] were also significantly related to the dependent variable. On the basis of these analyses it was therefore not possible to unambiguously identify the contribution of the Group factor to the variance in the RTs for incongruent trials.

A clearer result was obtained when the Conflict Effect was chosen as the dependent variable. The same covariates as before were included in the model. Group was a significant variable in the model, with a strong effect size [*F*(6,76) = 25.65, *p* < 0.001, η*^2^_*p*_* = 0.40]. None of the other covariates were significant. The smallest Conflict Effect was found among the CBLs (21 ms), followed by the TBLs (40 ms), and the largest one among the MLs (62 ms). Bonferroni *post hoc* analyses revealed that all groups were significantly different from each other: TBLs and CBLs (*p* = 0.006); TBLs and MLs (*p* = 0.001) and CBLs and MLs (*p* < 0.001). These results are also significant after correcting the criterion for significance for multiple comparisons using the Bonferroni correction (0.05/3 = 0.017). Further details about the adjusted mean RTs are given in Table 7.

**TABLE 7:** Conflict Effect, with means adjusted for the effect of the covariates.

1 = UK based Turks; 2 = UK based Cypriots	Mean	Std. Error	95% Confidence Interval	
			Lower Bound	Upper Bound
1	40.270^*a*^	3.867	32.575	47.965
2	21.478^*a*^	3.994	13.530	29.426
3	62.233^*a*^	3.790	54.690	69.776

*^*a*^Covariates appearing in the model are evaluated at the following values: Age = 30.10; Education = 3.18; Working Memory = 0.00; Non-verbal reasoning = −0.07.*

A very similar model was obtained with the Proportional score as the dependent variable Again there was a strong main effect of Group [*F*(2,80) = 25.48, *p* < 0.001, η*^2^_*p*_* = 0.39]. Education was marginally significant too, but with a very small effect size [*F*(1,80) = 3.97, *p* = 0.05, η*^2^_*p*_* = 0.05]. Bonferroni *post hoc* analyses revealed again that all groups (TBLs and CBLs, *p* = 0.008; TBLs and MLs, *p* < 0.001; CBLs and MLs, *p* < 0.001) were significantly different from each other. These results were also significant after correcting for multiple comparisons (0.05/3 = 0.017).

To test the robustness of the effects in analyses where all participants were closely matched (Czapka et al., 2020), we carried out a second series of analyses in which informants from all three groups were matched on all variables listed in Table 2, including the ones used as covariates in the first series of analyses. The only variable for which the two bilingual groups could not be matched is self-reported language mixing. In these analyses there was therefore only one independent variable (Group) and there were no covariates. All the analyses from the first series were repeated with very similar results. We ran a repeated measures ANOVA with Congruence as the within groups variable (two levels: RTs for congruent and incongruent trials) and Group as the between groups variable. There was a significant main effect of Congruence [*F*(1,28) = 91.50, *p* < 0.001], but no main effect of Group. There was a significant interaction between Group and Congruence [*F*(2,28) = 7.87, *p* = 0.002, η*^2^_*p*_* = 0.36].

The first follow-up univariate analysis, with Congruent RTs as the dependent variable, did not reveal a main effect of Group [*F*(3,28) = 2.03, *p* = 1.55, η*^2^_*p*_* = 0.13]. The second model with incongruent RTs as the dependent variable and Group as the independent variable was not significant either [*F*(3,28) = 1.06, *p* = 0.36, η*^2^p* = 0.07].

However, the model with the Conflict Effect as the dependent variable did reveal a main effect of Group [*F*(3,28) = 7.87, *p* < 0.001, η*^2^_*p*_* = 0.36].^10^ Bonferroni *post hoc* analyses showed that TBLs and CBLs were significantly (*p* = 0.009) different from each other, and also CBLs and MLs (*p* < 0.001). These differences remain significant after correcting for multiple comparisons. TBLs and MLs were not significantly different, although there was a slight tendency toward significance (*p* = 0.083).

Finally, we ran a model with the Proportional Score as the dependent variable. Again there was a main effect of Group [*F*(3,28) = 7.61, *p* = 0.002, η*^2^_*p*_* = 0.35]. The Bonferroni *post hoc* analysis shows that this time only the difference between CBLs and MLs was significant (*p* < 0.001).

In summary, the analyses presented here show that there was indeed a significant difference in Inhibitory Control between the monolinguals and the bilinguals, after controlling for the effect of covariates. This was most clearly seen in the Conflict Effect, i.e., the measure of inhibitory performance. In an ANCOVA with the Conflict Effect as the dependent variable and four covariates (with all 87 informants), the effect size of Group was reasonably strong (η*^2^_*p*_* = 0.40), and none of the covariates were significant. *Post hoc* analyses revealed that all groups were significantly different from each other, even after correcting for multiple comparisons. The Conflict Effect was greatest for MLs, and smallest for CBLs, while TBLs occupied the middle position, which means the CBLs demonstrated better inhibitory performance. The same rank order for the groups was found for the Proportional Score. These results were largely confirmed after closely matching informants from the three groups on key non-linguistic and linguistic variables, which led to a reduction in the informants to 31. However, possibly due to lack of statistical power, in this second series of analyses not all intergroup differences in the Conflict Effect remained significant: CBLs were significantly different from both TBLs and MLs, but TBLs and MLs were not significantly different after correcting for multiple comparisons.

### Explaining Variance in EFs Performance: The Role of Non-linguistic and Linguistic Variables

We used multiple regression to establish which variables explained the variance in EF performance. In this section we first report the results for the three groups taken together, and then for the monolinguals and bilinguals separately.

In analyses of all 87 informants the Conflict Effect was found to correlate weakly with Education (*r*_*s*_ = 0.22, *p* = 0.042) but not with other variables. Stronger correlations were found between the mean RTs of incongruent trials (IncongRTm) and key non-linguistic variables, which is why we decided to use this variable as the dependent variable for further analyses. IncongRTm was significantly correlated to three of the four key non-linguistic variables (see Table 8); only Working Memory correlated very weakly and non-significantly with IncongRTm. In other words, longer RTs were found in incongruent trials among older informants and those with higher education levels, but shorter RTs were found among informants with higher Non-verbal reasoning and Working Memory scores.

**TABLE 8:** Correlations between RTs from the Flanker task and non-linguistic variables.

	Spearman’s ρ	Age	Education	Non-verbal reasoning	Working memory
conflictRTs	*r*_S_	0.153	0.218*	−0.129	−0.143
	*p*	0.157	0.042	0.235	0.187
CongrRTm	*r*_S_	0.357**	0.140	−0.311**	−0.081
	*p*	0.001	0.197	0.003	0.457
IncongRTm	*r*_S_	0.466**	0.314**	−0.343**	−0.195
	*p*	0.000	0.003	0.001	0.071

*p < 0.05; **p < 0.01.

The non-linguistic variable which correlated strongest with IncongrRTm (Age) was entered in the first step in a hierarchical regression analysis, and other variables in subsequent steps (Non-verbal reasoning and Education). Only Age (β = 0.44) and Non-verbal reasoning (β = −0.25) but not Education were found to be significant predictors of IncongRTm. The overall ANOVA model was significant [*F*(2,84) = 17.61, *p* < 0.001, *R*^2^ = 0.28] and collinearity statistics (VIF = 1.03, Tolerance = 0.97) were within acceptable limits (Field, 2013) (see Table 9 for details).

**TABLE 9:** Linear model of predictors of IncongRTm among all informants (*N* = 87).

Model	*R*^2^	Adj *R*^2^	*SE*	*R*^2^ Change	*F* Change	*p* of *F* change
1	0.23	0.225	26.67	0.23	26.00	<0.001
2	0.30	0.279	25.74	0.06	7.29	<0.001

*Predictor model 1: Age; Predictors Model 2: Age and Non-verbal reasoning.*

In a separate analysis among the monolinguals, only Age (*r*_*s*_ = 0.61, *p* < 0.001) and Verbal Reasoning (*r*_*s*_ = −0.42, *p* = 0.02) correlated significantly with IncongRTm. These were subsequently entered in a hierarchical regression model, where only Age (β = 0.62) turned out to be a significant predictor of IncongRTm. While the addition of non-verbal reasoning (β = −0.25) led to a small increase in *R*^2^, this addition was not significant. The ANOVA model [*F*(1,28) = 17.15, *p* < 0.001] with Age as the sole predictor was significant and explained 36 percent of the variance in IncongRTm (see Table 10).

**TABLE 10:** Linear model of predictors of IncongRTm among monolinguals (*N* = 30).

Model	*R*^2^	Adjusted *R*^2^	*SE*	Change Statistics		
				*R*^2^ Change	*F* Change	*p*
1	0.380	0.358	29.56	0.38	17.15	0.001
2	0.440	0.399	28.60	0.06	2.91	0.100

*Predictor model 1: Age; Predictors Model 2: Age and Non-verbal reasoning.*

Subsequently, we ran several hierarchical regression models for bilinguals only. Our key aim was to establish to what extent linguistic and cultural identity variables would predict any variance in IncongRTm over and above the variance explained by non-linguistic variables. Therefore, we first entered three non-linguistic covariates into the model (Education, Non-verbal reasoning and Working memory)^11^. In a second step, we added a linguistic variable (reported language mixing) and two cultural variables (Hybrid and Alternating Identity Styles) which correlated most strongly with IncongRTm (see Table 11 for details). Code-mixing as measured with the CSFT did not correlate with IncongRTm, so was not included. The first model was significant [*F*(3,53) = 4.68, *p* < 0.001, Adj *R*^2^ = 0.17]. Only Non-verbal reasoning (β = −0.29, *p* = 0.03) and Working Memory (β = −0.31, *p* = 0.02) were significant predictors (see Table 12). The second model was significant too [*F*(5,51) = 11.63, *p* < 0.001, *R*^2^ = 0.49] and clearly explained far more variance. In this model, Non-verbal reasoning (β = −0.45, *p* < 0.001), Education (β = −0.27, *p* = 0.02), Hybrid Identities (β = −0.44, *p* = 0.03) and Alternating Identities (β = −0.97, *p* < 0.001) were significant predictors. Reported mixing was not a significant predictor (β = −0.09, *p* = 0.46).

**TABLE 11:** Spearman correlations between IncongRTm and non-linguistic and linguistic variables among bilinguals (*N* = 57).

	Age	Edu	NVR	WM	Esr	Tsr	MixR	HIS	AIS	EyU	TyU	TWL	EWL	CSFT
IncongRTm	0.268*	0.10	−0.32*	−0.36**	−0.07	0.08	0.457**	−0.492**	−0.60**	0.06	0.31*	0.23	−0.14	0.07
Age	1.00	0.28*	−0.22	−0.52**	−0.27*	0.58**	0.629**	−0.22	−0.34**	0.56**	0.92**	0.58**	−0.52**	0.00
Edu		1.00	−0.10	−0.12	0.04	0.27*	0.11	0.04	−0.21	0.04	0.24	0.20	−0.25	0.02
NVR			1.00	0.269*	0.03	−0.15	−0.30*	0.28*	0.03	−0.20	−0.26	−0.14	0.20	0.22
WM				1.00	0.14	−0.18	−0.52**	0.34**	0.46**	−0.43**	−0.49**	−0.47**	0.23	0.01
Esr					1.00	−0.56**	−0.31*	−0.05	0.10	0.24	−0.27*	−0.32*	0.49**	0.12
Tsr						1.00	0.48**	−0.04	−0.16	−0.10	0.60**	0.56**	−0.71**	−0.15
MixR							1.00	−0.41**	−0.52**	0.28*	0.65**	0.54**	−0.49**	0.02
HIS								1.00	0.72**	−0.25	−0.27*	−0.20	0.08	0.06
AIS									1.00	−0.13	−0.36**	−0.290*	0.16	−0.09
EyU										1.00	0.45**	0.20	−0.11	0.07
TyU											1.00	0.541**	−0.53**	0.05
TWL												1.00	−0.74**	−0.16
EWL													1.00	0.14

**TABLE 12:** First Linear Model of predictors of IncongrRTm, bilinguals only (*n* = 57).

Model	*R*^2^	Adjusted *R*^2^	*SE*	Change Statistics				
				*R*^2^ Change	*F* Change	df1	df2	Sig. *F* Change
1	0.2	0.17	22.19	0.21	4.68	3	53	<0.001
2	0.53	0.49	17.39	0.32	17.63	2	41	<0.001

*Model 1 Predictors: (Constant), Education, Working Memory, Non-verbal reasoning. Model 2 Predictors: (Constant), Education, Working Memory, Non-verbal reasoning, reported language mixing, Hybrid and Alternating Identity styles.*

**FIGURE 2:**
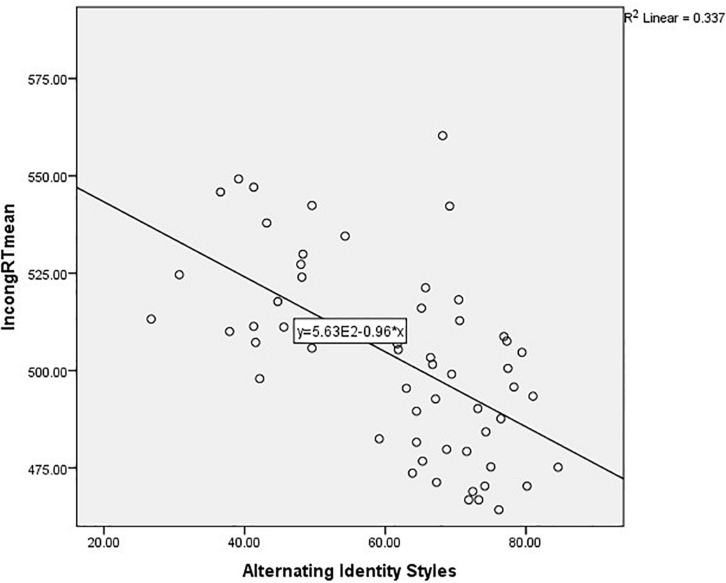
Regression line illustrating the relationship between Alternating Identity Styles and IncongRTmean.

Finally, we wanted to establish to what extent each of the two cultural identity variables were responsible for the additional explained variance in model 2. We therefore ran a hierarchical regression in which we separated the non-linguistic variables (step 1) from the Alternating Identities (step 2) and Hybrid Identities (step 3). Both the model with only Alternating Identities [*F*(3,53) = 12.03, *p* < 0.001] and the model with Alternating as well as Hybrid Identities [*F*(5,51) = 11.63, *p* < 0.001] were significant (see Table 13), but the *R*^2^ change associated with Alternating Identities (0.27) was much larger than the one associated with Hybrid Identities (0.05). The multicollinearity statistics were within acceptable limits (largest VIF = 4.41, Tolerance = 0.23). The relationship between Alternating Identities and IncongRTm is illustrated in Figure 2.

**TABLE 13:** Second Linear Model of predictors of IncongrRTm, bilinguals only (*n* = 57).

	*R*^2^	Adjusted *R*^2^	*SE*	*R*^2^	*F* Change	df1	df2	Sig. *F* Change
1	0.22	0.16	22.27	0.22	3.64	4	52	0.01
2	0.49	0.43	18.26	0.27	26.30	1	51	0.00
3	0.54	0.48	17.49	0.05	5.59	1	50	0.02

*Model 1 Predictors: (Constant), Education, Working Memory, Non-verbal reasoning. Model 2 Predictors: (Constant), Education, Working Memory, Non-verbal reasoning, reported language mixing, Alternating Identity styles. Model 3 Predictors: (Constant), Education, Working Memory, Non-verbal reasoning, reported language mixing, Alternating and Hybrid Identity styles.*

In summary, we have seen that only Age and Non-verbal reasoning were significant predictors of IncongRTm when all informants were considered together, explaining 28 percent of the variance in the dependent variable. In monolinguals, only Age was a significant predictor, which predicted 36 percent of the variance. In bilinguals, neither the CSFT nor reported code-mixing explained variance in EFs in the study, which was unexpected given our hypotheses. By contrast, the two multicultural identity variables, AIS and HIS, explained 32% of additional variance over and above Education, Working Memory and Non-verbal reasoning (overall explained variance 49%). The data clearly showed that the *R*^2^ change associated with Alternating Identities (0.27) was much larger than the one associated with Hybrid Identities (0.05). Thus, the data provide strong evidence for the impact of multicultural identity on Inhibitory Control.

## Discussion

This paper set out to disentangle the effects of managing two different languages and cultures on EFs in two groups of bilinguals by keeping the languages and immigration status constant, whilst varying the cultural backgrounds of the groups. The first group consisted of Turkey-born bilinguals (TBLs, *n* = 29), and the second one of Cyprus-born bilinguals (CBLs, *n* = 28), all of whom were first generation immigrants to the United Kingdom and spoke Turkish as their L1 and English as their L2. We first investigated differences in the code-switching habits and multicultural identities of each group and then investigated the differences in Inhibitory Control between the two groups and a group of monolingual speakers of English (ML, *n* = 30). Finally, we investigated the contribution of linguistic and non-linguistic variables to variance in Inhibition using regression analyses.

We developed a code-switching frequency task (CSFT) with examples of four different types of Turkish–English intrasentential code-switching to measure between group differences in this variable. As predicted on the basis of Muysken’s (2013) model of intrasentential code-switching, the CBLs were found to engage marginally more frequently than the TBLs in a form of code-switching which involves interactions between the lexica and the grammars of two languages (congruent lexicalization), but the groups did not differ with respect to other types of intrasentential code-switching. There was thus some support for our hypothesis that CBLs would engage more in congruent lexicalization, although the effect size was very small.

The existence of between groups differences regarding self-reported intrasentential code-switching reveals the complexity of obtaining valid information about informants’ codes-witching practices with a questionnaire. According to the questionnaire results, CBLs claimed to engage in “language mixing” significantly less often than TBLs. This difference was highly significant with a strong effect size. Because of the stigma attached to language mixing among Turkish-English bilinguals (Koban, 2013, 2016), we assume that CBLs underreport this behavior, and that the self-reported scores reflect attitudes rather than frequencies. The answer to the question why CBLs underreport language mixing by comparison with TBLs may be sought in CBLs’ stronger allegiance to the British way of Life (as reflected in answers to the LHQ). In the English-speaking world, code-switching is often seen as a sign of laziness, impure language use, or bad manners (Garrett, 2012; Jaworska and Themistocleous, 2018), and CBLs may have internalized these norms more than TBLs, due to the depth of language contact between English and Turkish in the history of Cyprus. Until it became independent in 1960, Cyprus was a colony of the United Kingdom, and English is very much present in the linguistic landscape and everyday life (Themistocleous, 2018). The existence of *negative* correlations between reported language mixing and the identity variables provides further evidence for the fact that answers to the question about language mixing are at least in part influenced by respondents’ attitudes or identity profiles.

In addition to the differences in reported language mixing, the most important differences between both groups resided in their multicultural identity profiles, which were measured with Ward et al.’s (2018) Multicultural Identity Styles Scale. According to Ward et al. (2018), bilinguals use different strategies to cope with intercultural differences: either they blend different elements from each culture (the hybrid identity style, HIS) or they keep both identities separate and alternate between different identities (the alternating identity style, AIS). CBLs and TBLs differed strongly in their answers to statements measuring these constructs, in that CBLs expressed stronger affinities with statements measuring Hybrid Identities (as we had predicted) but they also had more pronounced Alternating identities than TBLs, which we had not foreseen. While it is beyond the scope of the current paper to explain the reasons for these differences in any depth, we suggest these might be interpreted in the light of Tajfel’s (1981) social identity theory, according to which an individual’s social identity is derived from their membership of a social group together with the value and emotional significance attached to that membership. A possible reason behind the higher scores of CBLs on the MISS is *identity threat*, which according to Branscombe et al. (1999), is one of the key drivers of the dynamics of social identity processes. Because only one in three inhabitants of Cyprus lives in the North, the Turkish Cypriots are a minority in Cyprus and they feel threatened in their social identity by both the Greek Cypriots and the “settlers” from the Turkish mainland (Cakal, 2012). The latter constitute the majority group in their own country as well as among the Turkish immigrants in the United Kingdom and may therefore experience lower levels of identity threat. The difference in perceived threats posed by the other group might explain why in our study the CBLs claim to be less appreciative of the Turkish way of Life and more attached to the British way of Life than the TBLs. How exactly identity issues translate into behaviors, including attempts to balance or integrate one’s cultural identities by adopting strategies of alternating versus blending in everyday life (Ward et al., 2018) cannot be explored in depth in this paper. Nevertheless, the most surprising finding of the current paper was that these different strategies turned out to be relevant for participants’ scores on the Flanker task.

The results of the Flanker task revealed that the smallest Conflict Effect (differences in RTs between congruent and incongruent trials of the Flanker task) was found among CBLs, and the largest among the monolinguals, with the TBLs’ performance falling in between these two extremes. These intergroup differences were significant after controlling for Age, Education, Working Memory and Non-verbal reasoning. Thus, there was substantial evidence for our hypothesis that the bilingual groups would outperform monolinguals on tasks measuring Inhibition, and that the CBLs would outperform the TBLs on this task. Importantly, the CBL group engaging in more congruent lexicalization showed a reduced Conflict effect, which is in line with the findings of Hofweber et al. (2016) for bilinguals speaking typologically related languages. Hence, we assume congruent lexicalization amongst typologically distant languages also trains EFs and conflict monitoring.

We subsequently explored correlations between the Flanker task, code-switching and identity variables. Contrary to our expectations, the results on the CSFT did not correlate with the scores on the Flanker task, but there were mid strength correlations between *reported* intrasentential code-switching behavior, as measured by the questionnaire, and RTs on Incongruent trials (IncongRTm) on the Flanker task. In other words, those who claimed to mix languages more, needed more time to press the answer button for incongruent trials. These results could be interpreted as providing some support for Green and Abutalebi’s (2013) ACH, according to which engaging in “dense code-switching” does not recruit inhibitory control to the same extent as functioning in dual control modes whereby bilinguals switch between sentences but not within sentences. However, the absence of correlations between the results of the CSFT and reported language mixing makes it likely that these two tasks measured different constructs. As explained in detail in Hofweber et al. (2019), the CSFT offers respondents authentic examples of intrasentential code-switching, and is thus more likely to offer a valid reflection of bilinguals’ code-switching practices than a generic statement from a questionnaire that respondents might interpret in very different ways. Moreover, it is likely that the questionnaire scores were confounded by participants’ attitudes. Given the low validity of self-reported code-switching, the observed negative correlation therefore provides limited insights into the true relationship between code-switching and EFs.

In subsequent regression analyses, we regressed IncongRTm on a range of non-linguistic and linguistic variables. We found, first of all, that for all respondents taken together Age and Non-verbal reasoning were the key predictors. In a second, separate analysis of the monolinguals, Age was the only significant variable. In the third series of regressions, among bilinguals, we found that contrary to our predictions, intrasentential code-switching as measured with the CSFT did not explain any variance in the Flankers task results. It was particularly surprising that congruent lexicalization did not explain EF performance variance. The absence of a correlation between congruent lexicalization and EFs could be accounted for by the low frequency scores for congruent lexicalization. The low scores were possibly due to the typological distance between the languages, which means that there are few cognates and divergence between grammatical structures, reducing the likelihood of congruent lexicalization. As a result, variability in congruent lexicalization was small, which made correlational analyses challenging. This resulted in the absence of robust evidence for congruent lexicalization being a predictor of EF performance in the regression analyses.

The novel finding from the current study was that, among bilinguals, multicultural identity (AIS and HIS) explained variance over and above the non-linguistic variables entered in the model (Education and Non-verbal reasoning), and above the variance explained by reported language mixing. Reported language mixing was not retained in a model in which HIS and AIS were included. The β values for both identity variables were negative, which means that bilinguals with high scores on either the AIS or the HIS had shorter RTs on incongruent trials of the Flanker task. As the coefficient for AIS was much stronger than that for HIS, it is in particular bilinguals who tried to keep both identities separate and alternate between different identities that obtained shorter RTs. Thus, although our hypothesis that those with higher levels of hybrid identity would outperform those with lower levels of hybrid identities at EFs tasks was partly confirmed, contrary to our predictions AIS was a stronger predictor than HIS. This could be the case because their continual practice with Frame switching leads biculturals to develop a heightened context sensitivity (West et al. (2017, p. 979). We assume that it is this heightened context sensitivity which gives biculturals an advantage over monolinguals during a Flankers task, which requires test takers to select the relevant cue amidst flanking distractors which need to be inhibited. Biculturals’ heightened context sensitivity can also explain the findings of Ye et al. (2016, p. 848), who found that it was only in the mixed culture context that proficient bilinguals had an advantage over non-proficient bilinguals in a Flankers task. Again, we would argue it is their experience with switching between cultural frames (or mixing these) in daily life that gives them this this advantage. The fact that AIS explained more variance in EFs might be interpreted as showing that Frame switching leads to more cognitive advantages than Hybridizing. Whether or not preferences for Hybridizing and/or Frame switching can also explain biculturals’ performance on other EF tasks is an open question. As Poarch and Krott (2019) point out, the Simon task induces conflict by a spatial–stimulus-response mismatch. It is therefore possible that context sensitivity is less relevant for this task than for the Flankers task. This, in turn, may help explain why sometimes no correlation is found between these two EF tasks. An analysis of the relationship between biculturals’ performance on the MISS on the one hand, and the Simon and the Flanker task on the other hand might throw new light on this issue.

In summary, these data provide strong evidence for the hypothesis that for the Turkish–English bilinguals under study, the key explanatory variable was culture rather than bilingualism. We believe it was possible to achieve this result, first of all because we kept the languages as well as immigrant status constant whilst allowing cultural identity to vary systematically between both groups, which was novel by comparison to that of other studies. Second, we opted for an individual differences approach to the study of culture, and measured culture not only at the group level, as is the case in most other studies reviewed in this article, but also at the level of the individual. Therefore, our results show that sociocultural variables need to be incorporated in models of bilingual speech processing, and respondents’ degree of multiculturalism needs to be taken into account in future studies of the bilingual advantage.

A limitation of the current study was that our analyses of bilinguals’ code-switching practices relied on a receptive task, for which respondents needed to indicate to what extent they encountered different types of code-switching in their environment. Although Hofweber et al. (2019) demonstrated that the results of their codes-witching frequency task correlated with respondents’ productive code-switching behavior, we do not know whether this is also the case for the current groups of bilinguals. Finding examples of congruent lexicalization between Turkish and English turned out to be difficult; this is possibly due to the typological differences between the languages, which makes such an intimate form of code-switching challenging. If other techniques had been used to collect information about respondents’ code-switching habits, the effect of code-switching would perhaps have been more visible. Moreover, existing socio-linguistic frameworks of code-switching strongly suggest that bilinguals’ identity profiles actually co-vary with different code-switching styles (Muysken, 2000; Bhatt, 2008). Hence, it is possible that different code-switching patterns are actually part of the “package” of hybrid and alternating identity styles, although our questionnaire did not reveal such correlations. It is possible that our experimental instruments have not been subtle enough to pick up on this co-variance. Future research should therefore investigate the potential co-occurrence of different code-switching patterns with Multicultural identity styles using more ecologically valid measures of code-switching.

Another limitation might be our choice of a high monitoring Flanker task. As shown in Hofweber et al. (2020), bilinguals excel at those aspects of cognitive control which are trained by their code-switching practices. They argue that bilinguals who frequently engage in congruent lexicalization receive training in pro-active monitoring, but those who mainly engage in alternation, may receive training in reactive monitoring, which could be measured with a low monitoring Flanker task (82% of congruent and 9% of incongruent trials). However, in the current study, a Flanker task with low as well as high monitoring blocks could not be administered due to time constraints. In future projects, researchers could consider including Flanker tasks with different monitoring levels to explore the relationship between code-switching habits and Inhibition in more depth. In addition, as Poarch and Krott (2019) point out, it would be highly beneficial for the field if tasks tapping EFs and data analysis procedures such as treatment of outliers were standardized to ensure comparability of results between studies.

Future studies could also focus on the link between EFs and bilinguals’ ability to switch between cultures by investigating to what extent bilinguals deploy different pragmalinguistic strategies, such as apology strategies, which are well known to differ widely between cultures. While some bilinguals might alternate between clearly distinct strategies and use these in single or dual language contexts (Green and Abutalebi, 2013), others might prefer hybrid strategies which are a blend of strategies from different cultures and which are used irrespective of the different contexts or in “dense switching contexts.” We hope studies which focus on such intercultural issues can throw further light on the complex interaction between linguistic and cultural factors in shaping bilinguals’ EFs.

## Data Availability Statement

The raw data supporting the conclusions of this article will be made available by the authors, without undue reservation.

## Ethics Statement

The studies involving human participants were reviewed and approved by Ethics Committee of the Department of English Language and Applied Linguistics. The patients/participants provided their written informed consent to participate in this study.

## Author Contributions

JT-D: research design and data analysis. ZO: data collection. JH and MK: research design. All authors contributed to the article and approved the submitted version.

## Conflict of Interest

The authors declare that the research was conducted in the absence of any commercial or financial relationships that could be construed as a potential conflict of interest.

## Footnotes

1 The third strategy, called integrating involves “forming connections between cultures by recognizing similarities and reconciling differences, thereby linking the cultures while still retaining their original forms” (West et al., 2017, p. 972). This will not be considered in the current paper, which focuses mainly on the difference between hybridization and frame switching.

2 West et al. (2017) argue that there are subtle differences between blending and hybridizing because they see blending as related to perceiving overlap between cultures, while they interpret hybridizing as emphasizing the individual’s active role in fusing their cultures and creating something new.

3 This example was translated from the first author’s German-Turkish database collected among Turkish speakers in Germany. It is classified as congruent lexicalization because of the presence of a homophonous diamorph (*Ağustos*/*August*) and a switch of a copula (*is*).

4 The author uses the spelling *hade*, but for reasons of clarity we use the standard spelling *haydi.*

5 In what follows we will use the terminology of Ward et al. (2018) rather than that of West et al. (2017) because the former developed a questionnaire, the Multicultural Identity Styles Scale (MISS), which makes it possible to measure individuals’ preferences for the two key strategies (hybridization/blending and Frame Switching/Alternating).

6 For the identification of outliers we accepted the analysis given by SPSS where four cases were identified as outliers in the boxplot. Among these four RTs two were extreme outliers in that they were higher than the 75th percentile (534.53 ms.) + 3 times the interquartile range (47.36), that is higher than 676.61 ms. Two were mild outliers in that they were higher than the 75th percentile + 1.5 times the IQR, that is higher than 605.57 ms.

7 https://blclab.org/lhq3/

8 We used non-parametric tests because the means of self-reported code-mixing behavior were not normally distributed. Log transforming the variable did not help to remedy this.

9 This was checked for all computations and found to be non-significant in all cases. For reasons of space this is not reported each time.

10 As there is only one independent variable, the *F*-value for the overall model is the same as that for the independent variable.

11 As the number of informants in a regression analysis should be ten times the number of variables (Hair et al., 2014) we left out Age, which was not significant.
